# The effects of anti-PD-L1 monoclonal antibody on the expression of angiogenesis and invasion-related genes

**DOI:** 10.55730/1300-0152.2661

**Published:** 2023-06-07

**Authors:** Cansu BABAHAN, Samira ABDI ABGARMI, Fatma Gizem SONUGÜR, Müge ÖÇAL, Hakan AKBULUT

**Affiliations:** 1 Ankara University Cancer Research Institute, Ankara, Turkiye; 2 Department of Medical Oncology, School of Medicine, Ankara University, Ankara, Turkiye

**Keywords:** Anti-PD-L1, E-cadherin, immunotherapy, metastasis, PD-L1, VEGFA

## Abstract

**Background/aim:**

The role of PD-L1 in regulating the immunosuppressive tumor microenvironment via its binding on PD-1 receptors is extensively studied. The PD-1/PD-L1 axis is a significant way of cancer immune escape, and PD-L1 expression on tumor cells is suggested as a predictive marker for anti-PD-1/PD-L1 monoclonal antibodies (MoAbs). However, the tumor-intrinsic role of PD-L1 is not known well. Therefore, we aimed to investigate the effects of anti-PD-L1 antibodies on the expression of angiogenesis and metastasis-related genes in tumor cells.

**Materials and methods:**

The experiments were done with prostate cancer and melanoma cells with low PD-L1 expression (<5%) and prostate and breast cancer cells with high PD-L1 expression (>50%). The gene and protein expressions of VEGFA, E-cadherin, TGFβ1, EGFR, and bFGF in tumor cells were assayed at the 3 different doses of the anti-PD-L1 antibody.

**Results:**

We found that VEGFA, E-cadherin and TGFβ1 expressions increased in PD-L1 high cells but decreased in PD-L1 low cells after anti-PD-L1 treatment. EGFR expression levels were variable in PD-L1 high cells, while decreased in PD-L1 low cells upon treatment. Also, the anti-PD-L1 antibody was found to increase bFGF expression in the prostate cancer cell line with high PD-L1 expression.

**Conclusion:**

Our results suggest that the binding of PD-L1 on tumor cells by an anti-PD-L1 monoclonal antibody may affect tumor-intrinsic mechanisms. The activation of angiogenesis and metastasis-related pathways by anti-PD-L1 treatment in PD-L1 high tumors might be a tumor-promoting mechanism. The decrease of VEGFA, TGFβ1 and EGFR upon anti-PD-L1 treatment in PD-L1 low tumor cells provides a rationale for the use of those antibodies in PD-L1 low tumors.

## 1. Introduction

Cancer immunotherapy is a strategy that provides elimination of tumor cells by restoring the ability of the host immune system to recognize and destroy the tumor (Ren et al., 2020). Tumor cells hijack the PD-1/PD-L1 interaction from the immune system to suppress the antitumor T-cell responses through PD-L1 expression, thereby escaping from immunosurveillance (Ciciola et al., 2020). Many solid tumors have been found to overexpress PD-L1, which is associated with a poor prognosis. PD-L1 expression has been suggested as a predictive marker for anti-PD-1 and anti-PD-L1 antibody treatments (Salmaninejad et al., 2019). Immunotherapies targeting the PD-1/PD-L1 checkpoint have been approved by the U.S. Food and Drug Administration to treat various cancers (Ju et al., 2020). However, in many tumors, including triple-negative breast cancer, prostate cancer, nonsmall cell lung cancer, renal cell cancer, and melanoma, PD-1/PD-L1 blockade therapy can provide durable responses in only 20% of patients (Wu et al., 2019).

Epithelial-mesenchymal transition (EMT) and angiogenesis are two critical components that support tumor growth and metastasis in the tumor microenvironment (Jiang et al., 2018). EMT, a process in which epithelial cells transform phenotypically into mesenchymal cells, lose their adhesive properties and cell-cell contacts and gain migration properties, has been shown to play an important role in cancer metastasis ( Suda et al., 2017; Li et al., 2018). In addition, EMT, which is related to chemo-resistance and radio-resistance, plays an essential role in escaping tumor cells from the host immune system (Kurimoto et al., 2016). Angiogenesis, a process that involves the formation of new blood vessels from preexisting blood vessels, promotes the growth and metastasis of the solid tumor (Ramjiawan et al., 2017; Yi et al., 2019). In several studies, angiogenesis has been found to play a significant role in suppressing the immune system and thus leads to the development of primary and secondary resistance to immunotherapy (Jenkins et al., 2018; Yi et al., 2019). VEGF contributes to the escape of tumor cells from the immune system and is suggested as an immunosuppressive cytokine (McDermott et al., 2018; Liu et al., 2019). bFGF, which is overexpressed in many types of cancer, can trigger the formation of metastatic tumor phenotype by promoting the progression of tumor cells and increasing its angiogenic potential (Korc and Friesel, 2009; Akl et al., 2016). TGF-β, a pleiotropic cytokine associated with poor prognosis in many tumor types, is thought to play a protumorigenic role by promoting immunosuppression, angiogenesis, metastasis, tumor cell EMT and tumor immune escape in advanced cancers (Knudson et al., 2018; Mariathasan et al., 2018). EGFR is overexpressed in many cancer types and promotes cell proliferation and differentiation, apoptosis, angiogenesis, and metastasis-related signal pathways (Sasada et al., 2016; W. Zhang et al., 2017). E-cadherin expression, which has tumor suppressor effects, has been shown to decrease during tumor development in many cancers (French et al., 2002). Loss of E-cadherin expression, an epithelial cell marker, is one of the main features of EMT (Li et al., 2018).

The PD-1/PD-L1 axis is mainly involved in tumor cell-host immune system interactions. PD-L1 monoclonal antibodies exert their primary efficacy by binding PD-L1 receptors, thus preventing PD-1 binding. The immune resistance mechanisms involve different sides, including tumor cells, immune cells, and other cells in the tumor microenvironment. However, the PD-L1 receptor also acts as an antiapoptotic molecule on cancer cells (Ghebeh et al., 2010). Likewise, the PD-L1 signaling of tumor cells might also be involved in upregulating tumor-promoting mechanisms such as stemness properties, EMT and angiogenesis (Dong et al., 2018). The effect of PD-L1 expression level in tumor cells on angiogenesis and metastasis is not well known. There are conflicting results regarding the correlation between PD-L1 and proangiogenic factor expression levels (Sasada et al., 2016; Jenkins et al., 2018). The primary aim of the current study is to study the effects of anti-PD-L1 blockade only on the tumor cell side. In the current study, we investigated the expression levels of genes related to tumor angiogenesis and metastasis in PD-L1 low and high expressing tumor cells and the effects of anti-PD-L1 treatment on the expression of those genes.

## 2. Materials and methods

### 2.1. Cell culture

Human breast cancer cell lines (MDA-MB-231, MCF-7, and MDA-MB-468), human prostate cancer cell lines (LNCaP and DU145), human colon cancer cell line (HT-29), human lung cancer cell line (A-549), and human melanoma cell lines (A-375, G-361, WM115, and MDA-MB-435) were obtained from the American Type Culture Collection (ATCC, USA). While the MCF-7, A-375, A-549, MDA-MB-435, and G-361 cell lines were maintained in Dulbecco’s Modified Eagle Medium (BI, Israel); the WM115 and MDA-MB-468 cell lines in Eagle’s Minimum Essential Media (BI, Israel); the LNCaP, MDA-MB-231, and DU145 cell lines in Roswell Park Memorial Institute-1640 (Sigma Aldrich, USA) and the HT-29 cell line in McCoy’s 5A medium (Lonza, Switzerland) supplemented with 10% fetal bovine serum (BI, Israel).

### 2.2. Evaluation of PD-L1 expression on tumor cells by flow cytometer

The PD-L1 expression on tumor cells was assayed with flow cytometry. After washing with phosphate-buffered saline (BI, Israel), the cells were harvested by enzyme-free cell dissociation buffer (Thermo Fisher, USA). The harvested cells (1.5 × 10^6^) were stained with phycoerythrin (PE)-conjugated anti-PD-L1 antibody (Biolegend, USA), and the PD-L1 expressing cells were quantified by Navias Flow Cytometer (Beckman Coulter, USA).

### 2.3. Cell proliferation assay and determination of working concentrations of anti-PD-L1 MoAb

We used an anti-PD-L1 antibody to target the PD-L1 pathway in tumor cells. To determine the working concentrations of the antibody, we first studied the cytotoxic effects of the anti-PD-L1 antibody on tumor cells by MTT assay (Amresco, USA). Briefly, a total of 5 × 10^3^ cells were plated in 200 mL medium in each well of a 96-well plate. After 24 h, anti-PD-L1 monoclonal antibody (MoAb) (Avelumab, Merck, Switzerland) was diluted to the nine different (160, 80, 40, 20, 10, 5, 2.5, 1.25, and 0.625 μg/mL) concentrations in the test medium and added as treatment in triplicate for 72 h. The MTT assay was repeated thrice for each cell line to determine the half-maximum inhibitory concentration (IC50).

### 2.4. RNA isolation and quantitative real-time polymerase chain reaction (qPCR)

The total RNA isolation and cDNA synthesis were performed with commercial kits (Qiagen, Germany). Specific primers and probes for VEGFA, E-cadherin, EGFR, TGFβ1, bFGF and β-actin were designed and checked with Primer3web (version 4.0.0), Primer-Blast, NCBI-Blast. The primers and TaqMan probes used for qPCR are listed in Supplementary Table 1. The mRNA expression by qPCR was repeated thrice for each cell line. The qPCR amplification was performed using a 1X qPCR Mix (Solis Biodyne, Estonia). The qPCR profile consisted of 10 min initial denaturation at 95 °C, followed by 40 cycles (95 °C for 20 s, 60 °C for 1 min). qPCR was conducted on the Bio-Rad C1000 Thermal Cycler CFX96 Real-Time System (Bio-Rad, USA). Delta-delta Ct method (2^−ΔΔCt^ method) was used for gene expression analysis in cells.

### 2.5. Protein expression analysis

Cell lysates were prepared by using a mammalian cell lysis kit (Sigma, USA). The protein expression by western blotting was repeated thrice for each cell line. The following primary antibodies; polyclonal VEGFA, bFGF, TGFβ1 and E-cadherin (Abcam, UK), beta Actin (Bioss, USA) and EGFR (Santa Cruz, USA) were used. Following the peroxidase-conjugated secondary IgG (H + L) (Jackson Immuno Research, USA), immune complexes were visualized using luminol (Bio-Rad, USA). The chemiluminescence from the specific bands was visualized by Molecular Imager GelDoc XR+ ChemiDoc XRS+ Imaging System (BioRad, USA). The expression ratios of VEGFA, E-cadherin, TGFβ1, EGFR, bFGF, and β-actin from the blots were analyzed using Molecular Imager GelDoc XR+ ChemiDoc XRS+ Imaging System and Image Lab Software 6.0.1 (Bio-Rad, USA). After determining the intensities of the obtained bands, normalized protein fold ratios were calculated by proportioning the intensity value of each band to the corresponding β-actin band for each dose level.

### 2.6. Measurement of cytokine levels

VEGFA, E-cadherin, TGFβ1, EGFR, and bFGF concentrations in the cell culture supernatants, repeated thrice for each cell line, were measured using a Human VEGFA, E-cadherin, TGFβ1, EGFR, and bFGF ELISA Kit (FineTest, China) according to the manufacturers’ instructions and OD values were measured at 450 nm using a microplate reader (Thermo Scientific Multiskan GO, USA).

### 2.7. Statistical analysis

The data were expressed as means ± standard deviations (SD) and were analyzed using one-way ANOVA, Student’s t, and Spearman’s correlation test (SPSS 20.0). A p-value of less than 0.05 (<0.05) was considered significant.

## 3. Results

### 3.1. PD-L1 expression levels on tumor cells

The PD-L1 expression level of tumors were determined by measuring median fluorescence intensity (MFI) on flow cytometry. The normalized MFI values (MFI of cells stained with anti-PD-L1 MoAb/MFI of cells stained with isotype control) of tumor cells that differed markedly in the histogram for positive cells were calculated. (Supplementary Figure 1). The cell lines were divided into two groups: low PD-L1 expression having less than 10-fold change MFI (MDA-MB-435 and DU145 cells) and high PD-L1 expression having more than 10-fold change MFI (LNCaP and MDA-MB-231 cells) used in the study.

### 3.2. The anti-PD-L1 MoAb did not cause toxicity on cancer cell lines

The cytotoxic effect of the anti-PD-L1 MoAb was found to be very low within the range of 0.625–160 μg/mL doses of the drug. The viability of the cells was over 90% at all studied dose levels of anti-PD-L1 antibody (Supplementary Figure 2). Therefore, we choose 40 μg/mL, 20 μg/mL, and 2 μg/mL doses of the anti-PD-L1 MoAb, which cover the corresponding blood levels of the drug in humans following intravenous (IV) injection of a routine dose of 800 mg every two weeks. Selected doses for anti-PD-L1 MoAb were also used in experiments with IgG1 isotype control antibody (BioLegend, USA). Although the in vitro doses do not correspond to the in vivo dosing, we aimed to test the effects of stable doses, covering the maximum blood levels of the patients, in the current study.

### 3.3. The anti-PD-L1 MoAb affects cytokine levels and gene and protein expressions in PD-L1 high tumor cells

**Figure 1:**
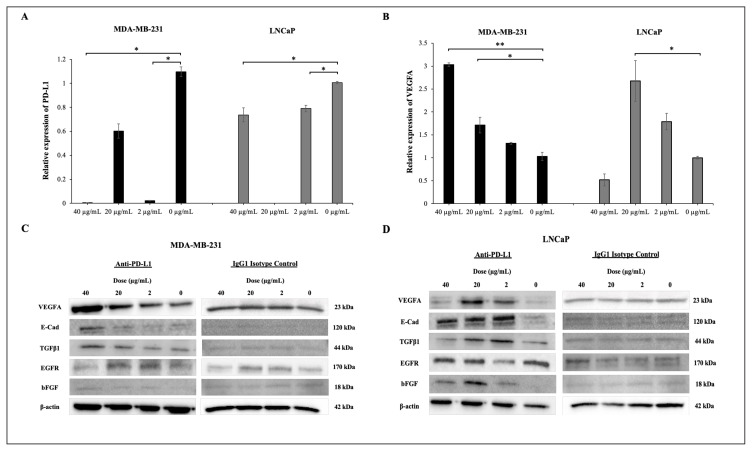
PD-L1 (A) and VEGFA (B) gene expressions at 72 h after anti-PD-L1 antibody treatment in tumor cells with high PD-L1 expression levels (*p ≤ 0.05 **p ≤ 0.01). Protein expressions at 72 h after antibody treatment in tumor cells with high PD-L1 expression levels; (C) MDA-MB-231 and (D) LNCaP cells.

The tumor cells were plated with 5 × 10^5^ cells/well in 6-well plates with three replicates for each MoAb dose, IgG1 isotype control and negative control. Following the incubation of the cells at 37 °C and 5% CO_2_ for 24 h, three different doses (40 μg/mL, 20 μg/mlL, and 2 μg/mL) of anti-PD-L1 antibody and IgG1 isotype control were applied to four cell lines (MDA-MB-231, LNCaP, DU145 and MDA-MB-435 cells). Cells were harvested to analyze gene expression by qPCR after 24 and 72 h and for analysis of protein expression by western blotting after 72 h. Supernatants of cells were collected for quantifying the secreted cytokines by ELISA after 72 h. At the 72nd hour of the treatment, a decrease in PD-L1 gene expressions was observed in both cells with high PD-L1 expression, in line with the expected treatment efficacy (Figure 1A).

#### 3.3.1. The anti-PD-L1 MoAb treatment increases VEGFA expression in PD-L1 high tumor cells

We found a decrease in VEGFA gene expression at 40 μg/mL (p = 0.03), 20 μg/mL and 2 μg/mL (p = 0.032) doses at the 24th hour of MoAb treatment applied to MDA-MB-231 cells, while an increase in VEGFA gene expression was observed at 40 μg/mL (p = 0.001), 20 μg/mL (p = 0.046) and 2 μg/mL doses at the 72nd hour of treatment. In LNCaP cells, VEGFA gene expression increased at 20 μg/mL (p = 0.019) and 2 μg/mL doses at the 72nd hour of treatment (Figure 1B). VEGFA gene expression in MDA-MB-231 cells treated with IgG1 isotype control was significantly decreased at doses of 40 μg/mL (p < 0.001) and 20 μg/mL (p < 0.001) at the 72nd hour of treatment. IgG1 isotype control treatment did not cause a change in VEGFA gene expression in LNCaP cells (Supplementary Table 2).

In addition, while VEGFA protein expression was significantly increased at all three doses at 72 h of anti-PD-L1 treatment, no clear change was observed in protein expressions after IgG1 isotype control in MDA-MB-231 cells. In LNCaP cells, at 72 h of treatment, a significant increase in VEGFA protein expression was detected at 20 μg/mL and 2 μg/mL doses, while IgG1 isotype control treatment had no clear effect on VEGFA protein expression (Figures 1C and 1D, Table 1).

In MDA-MB-231 cells, although there was an increase in the levels of secreted VEGFA at doses 40 μg/mL and 20 μg/mL at 72 h of anti-PD-L1 treatment, it was not statistically significant. Similarly, in LNCaP cells, the expression of secreted VEGFA increased at 20 μg/mL and 2 μg/mL doses, although not statistically significant (Table 2).

These results suggest that the anti-PD-L1 antibody treatment causes an increase in VEGFA expression in cells with high PD-L1 expression, independent of the IgG1 class effect.

#### 3.3.2. The anti-PD-L1 MoAb treatment increases E-cadherin expression in PD-L1 high tumor cells

**Figure 2:**
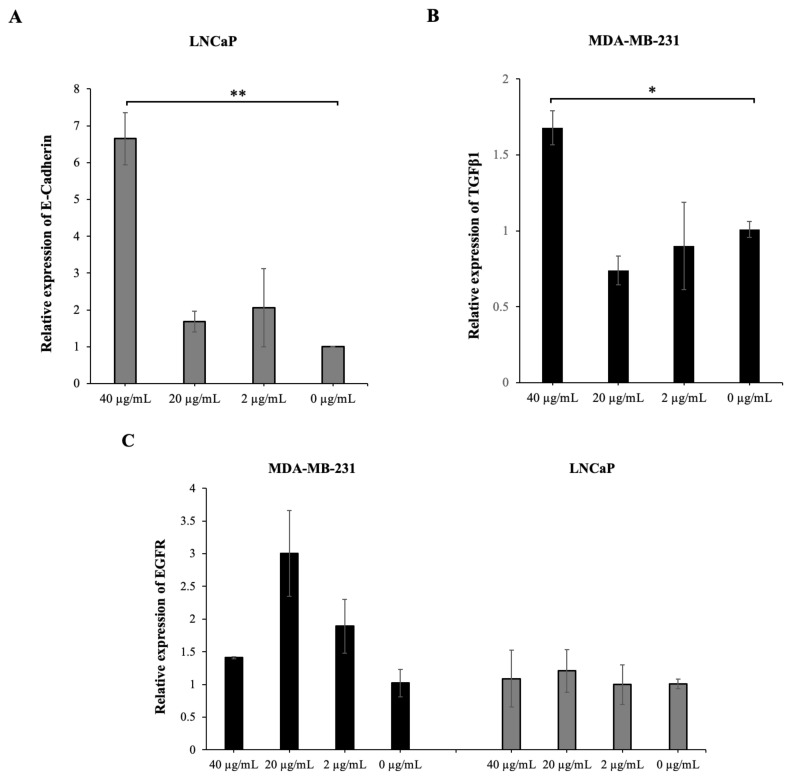
E-cadherin (A), TGFβ1 (B) and EGFR (C) gene expressions at 72 h after anti-PD-L1 antibody treatment in tumor cells with high PD-L1 expression levels (*p ≤ 0.05 **p ≤ 0.01).

At the 72nd hour of MoAb, the expression of E-cadherin in LNCaP cells treated at a dose of 40 μg/mL showed a significant increase compared to the level of E-cadherin expressed in untreated cells (p = 0.001) (Figure 2A), while no change was observed in E-cadherin gene expression after IgG1 isotype control treatment (Supplementary Table 2).

We could not detect E-cadherin expression in untreated (0 μg/mL) or IgG1 isotype control-treated MDA-MB-231 cells with qPCR and western blotting, which is consistent with previous reports (van de Wetering et al., 2001; Eslami Amirabadi et al., 2019). However, an increase in E-cadherin expression was observed in MDA-MB-231 cells following anti-PD-L1 treatment. At the 72nd hour of anti-PD-L1 treatment, the E-cadherin protein expression level increased significantly at the dose of 40 μg/mL. In LNCaP cells, while an increase in E-cadherin protein expression was observed in all three doses at the 72nd hour of treatment, no change was detected in protein expression after IgG1 isotype control treatment (Table 1).

After 72 h of incubation, the secreted E-cadherin levels increased significantly at the dose of 40 μg/mL (p = 0.044) in MDA-MB-231 cells and increased at all three doses, being statistically significant at the 40 μg/mL dose (p = 0.001) in LNCaP cells (Table 2).

These results from two different cell lines confirm that anti-PD-L1 antibody treatment causes an increase in E-cadherin expression in cells with high PD-L1 expression, independent of the IgG1 antibody effect.

#### 3.3.3. The anti-PD-L1 MoAb treatment increases TGFβ1 expression in PD-L1 high tumor cells

At the 72nd hour of the MoAb treatment of MDA-MB-231 cells, a significant increase of TGFβ1was observed at the 40 μg/mL dose (p = 0.046) (Figure 2B). However, a significant reduction of TGFβ1 occurred at the 40 μg/mL dose in MDA-MB-231 cells treated with IgG1 isotype control (p = 0.046) (Supplementary Table 2).

At the 72nd hour of the treatment, a significant increase in TGFβ1 protein expression was detected in MDA-MB-231 cells at a dose of 40 μg/mL of anti-PD-L1 antibody but not of isotype IgG1. TGFβ1 gene expression was not detected in LNCaP cells after anti-PD-L1 MoAb and IgG1 isotype control treatment. However, a significant increase in TGFβ1 protein expression was observed at the 20 μg/mL and 2 μg/mL doses at the 72nd hour of treatment. It has also been shown that IgG1 isotype control does not affect TGFβ1 protein expression in PD-L1 high tumor cells (Table 1).

At the 40 μg/mL MoAb treatment dose, an increase was observed in the level of secreted TGFβ1, although not significant in MDA-MB-231 cells. In LNCaP cells, at the 20 μg/mL and 2 μg/mL doses, an increase in secreted TGFβ1 level was observed although not significant (Table 2).

Our findings from two different cell lines suggest that anti-PD-L1 antibody treatment increases TGFβ1 expression in tumor cells with PD-L1 expression by more than 50%.

#### 3.3.4. The anti-PD-L1 MoAb treatment has varying effects on EGFR expression in tumor cells with high PD-L1 expression

In our study, at the 72nd hour of the treatment, the EGFR gene expression levels in both MDA-MB-231 cells and LNCaP cells were like the control groups (Figure 2C). After IgG1 isotype control treatment, EGFR gene expression was significantly increased at the 2 μg/mL dose in MDA-MB-231 cells (p = 0.001) and slightly increased at 20 μg/mL and 2 μg/mL doses in LNCaP cells (Supplementary Table 2).

At 72 h of anti-PD-L1 treatment, EGFR protein expression in MDA-MB-231 cells increased at 20 μg/mL and 2 μg/mL doses, but this increase was also found after the IgG1 isotype control and it was determined that EGFR protein level in LNCaP cells decreased significantly with anti-PD-L1 MoAb treatment, especially at dose 2 μg/mL; however, an increase was observed at dose 40 μg/mL after IgG1 isotype control (Table 1).

Similarly, the level of secreted EGFR in MDA-MB-231 cells increased at doses of 40 μg/mL (p = 0.019) and 20 μg/mL (p = 0.045) after treatment. Like EGFR protein expression in LNCaP cells, the level of secreted EGFR was found to be significantly reduced at 2 μg/mL dose (p = 0.013) (Table 2).

As a result, it is thought that the increase in EGFR in MDA-MB-231 cells after anti-PD-L1 MoAb treatment is not treatment specific, but rather an IgG1 antibody class effect.

#### 3.3.5. The anti-PD-L1 MoAb treatment increases bFGF expression in prostate cancer cells with high PD-L1 expression

bFGF gene expression was not detected in LNCaP cells after anti-PD-L1 Moab and IgG1 isotype control treatments, but at the 72nd hour of MoAb treatment, bFGF protein expression and secreted bFGF level in LNCaP cells increased significantly at 20 μg/mL dose (p = 0.005) (Tables 1 and 2). This finding suggests that anti-PD-L1 MoAb treatment may trigger bFGF expression in LNCaP cells.

### 3.4. The anti-PD-L1 MoAb treatment might have effects on angiogenesis and metastasis-related genes in PD-L1 low tumor cells

**Figure 3:**
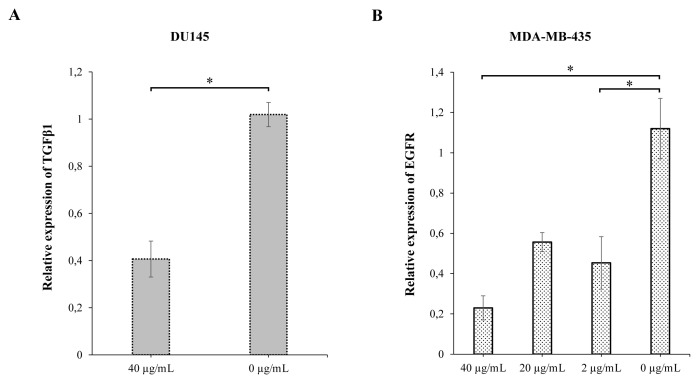
TGFβ1 (A) and EGFR (B) gene expressions at 72 h after anti-PD-L1 antibody treatment in tumor cells with low PD-L1 expression levels (*p ≤ 0.05).

We found that the TGFβ1 gene expression in DU145 cells decreased significantly at all doses at the 24th hour of MoAb treatment (p < 0.01) and at the 72nd hour of the treatment, no gene expression was detected in the 20 μg/mL and 2 μg/mL doses, and a decrease was detected in the 40 μg/mL dose (Figure 3A). At 72 h of MoAb treatment, EGFR gene expression in MDA-MB-435 cells decreased significantly at 40 μg/mL and 2 μg/mL doses (p = 0.04) (Figure 3B). The IgG1 isotype control did not affect TGFβ1 and EGFR gene expressions in DU145 cells. In DU145 cells, we found a significant decrease in VEGFA expression at all three doses following 24 h of treatment (p < 0.01). On the contrary, the IgG1 isotype control significantly increased the VEGFA gene expression at all three doses in DU145 cells (p < 0.01) (Supplementary Table 2).

Like qPCR results, TGFβ1 protein expression in DU145 cells at 72 h of MoAb treatment decreased at all three doses. After 72 h of treatment, VEGFA and E-cadherin protein expressions decreased significantly at doses of 40 μg/mL and 20 μg/mL.

**Figure 4:**
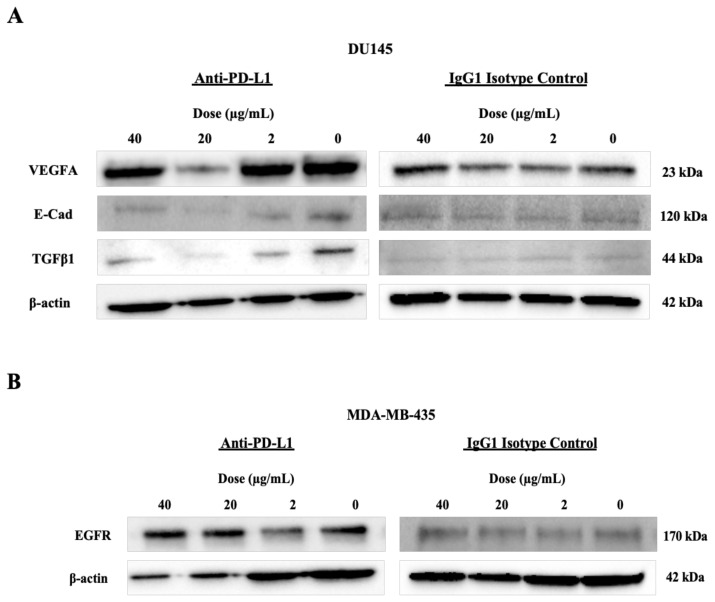
Protein expressions at 72 h after anti-PD-L1 and IgG1 isotypic antibody treatment in tumor cells with low PD-L1 expression levels; (A) DU145 and (B) MDA-MB-435 cells.

The IgG1 isotype control has no effect on VEGFA and E-cadherin protein expression in DU145 cells (Figure 4A, Table 3). The level of secreted TGFβ1, VEGFA and E-cadherin was significantly decreased at 20 μg/mL dose (p < 0.05) in DU145 cells (Table 4).

In MDA-MB-435 cells, similar to the qPCR result, decreased EGFR protein expression and secreted EGFR levels were observed at 72 h of treatment at the 2 μg/mL dose. No change was detected in the EGFR protein expressions after IgG1 isotype control in MDA-MB-435 cells (Figure 4B, Tables 3 and 4).

Looking at these results, it can be said that the decreased VEGFA, E-cadherin, TGFβ1 and EGFR expression in PD-L1 low tumor cells after treatment is specific to anti-PD-L1 MoAb treatment.

## 4. Discussion

Immune checkpoint inhibitors (ICIs) have yielded durable responses in various types of cancers. The binding of an anti-PD-L1 antibody to PD-L1 prevents the interaction between PD-L1 and the inhibitory T cell receptors PD-1 and B7.1, resulting in T cell-mediated, adaptive antitumor immune responses and T cell reactivation and cytokine production. Unlike atezolizumab and durvalumab, avelumab, a fully humanized IgG1 antibody, has a wild-type IgG1 crystallizable fragment (Fc) region compound to engage with Fc-γ receptors on natural killer cells and induce tumor-directed antibody-dependent cell-mediated cytotoxicity (ADCC) in preclinical studies. Therefore, avelumab utilizes adaptive and innate immune mechanisms to destroy cancer cells (Boyerinas et al., 2015; Akinleye and Rasool, 2019). However, clinical experience with ICIs has shown that those antibodies work only in a selected group of cancer patients. In common tumors such as breast and prostate cancer, most patients do not respond to ICIs (Bai et al., 2020).

The PD-1/PD-L1 axis is mainly involved in tumor cell-host immune system interactions. PD-L1 monoclonal antibodies exert their primary efficacy by binding PD-L1 receptors, thus preventing PD-1 binding. Apart from the effects of blocking PD-1/PD-L1 interaction on immune cell functions, there might be some changes in tumor cells upon binding the anti-PD-L1 antibody. Recent studies have shown that PD-L1 expression in tumor cells plays a critical role in promoting cell proliferation, migration, metastasis, resistance to therapy, and EMT in cancer (Alsuliman et al., 2015; Kim et al., 2016; Lou et al., 2016; Ock et al., 2016). PD-L1 receptor signaling is also a significant pathway for the tumor cell, an antiapoptotic way for tumor cells. Although the role of PD-L1 in the regulation of immunosuppressive tumor microenvironment via its binding on PD-1 receptors is extensively studied, recent studies reveal a distinct tumor-intrinsic role for PD-L1 in modulating epithelial-to-mesenchymal transition (EMT), cancer stem cell (CSC)-like phenotype, metastasis and resistance to therapy apart from its role in tumor immunology (Dong et al., 2018). Likewise, it is not well known how PD-L1 expression level in tumor cells plays a role in the response to ICIs and how proliferation and invasion processes progress following treatment. There needs to be more published data on the effects of PD-L1 blockade by monoclonal antibodies on tumor cell behaviors. Therefore, this paper will be a preliminary study focusing on the efficacy of binding PD-L1 by anti-PD-L1 monoclonal antibodies on tumor cells.

We could not find a precise dose-dependent effect of anti-PD-L1 antibody on PD-L1 expression. However, a tendency for a dose-dependent decrease in the expression of PD-L1 in both MDA-MB-231 and LnCaP cells was noticed. PD-L1 has a survival effect on tumor cells via decreasing apoptosis. Therefore, a possible inhibitory effect of anti-PD-L1 monoclonal antibody binding to PD-L1 might trigger the downregulation of the receptor itself. However, the current study’s design does not explain this mechanism and the discrepant results with the 2 μg dose on MDA-MB-231 cells.

The changes in tumor cells by binding PD-L1 receptors with anti-PD-L1 antibodies might induce tumor-promoting pathways in tumors. In the current study, we aimed to investigate the relationship between PD-L1 expression in tumor cells and various angiogenesis and metastasis-related gene expressions by targeting PD-L1 with a monoclonal antibody.

Proangiogenic factors are influential in the formation of an immunosuppressive microenvironment (Ciciola et al., 2020). Besides the well-known role of VEGF on angiogenesis, numerous studies have shown that VEGF can support the escape of tumor cells from the immune system by inhibiting dendritic cell maturation (Tamura et al., 2019). VEGF has also been shown to decrease T cell function and number, as it increases PD-L1 expression on dendritic cells (Bourhis et al., 2021). There are conflicting reports regarding the correlation between PD-L1 and VEGF expression in tumors. While a negative correlation between PD-L1 and VEGFA expression was reported in patients with lung cancer, a positive correlation was found in breast cancer patients (Sasada et al., 2016). We found a positive correlation between PD-L1 and VEGFA expression in the tumor cell lines including MDA-MB-231, LNCaP, MDA-MB-435, and DU145. However, the decreased expression of PD-L1 with the anti-PD-L1 treatment accompanied an increased VEGFA expression. This suggests that the combination of anti-PD-L1 antibody and anti-VEGFA therapy seems to be a reasonable option to increase therapeutic efficacy in tumors with high PD-L1 expression. Recent clinical studies show that anti-VEGFA drugs can increase antitumor immunity in human cancers by modulating immune cell compositions and enhancing immune responses to the tumor (Manzoni et al., 2010; Terme et al., 2013). In addition, it has been shown in various studies that anti-VEGF therapy increases the efficacy of immune checkpoint inhibitors in various cancer types (Allen et al., 2017; Meder et al., 2018). Interestingly, we found a significant decrease in VEGFA expression in tumor cells with low PD-L1 expression after anti-PD-L1 treatment. The results of in vitro experiments in the current study cannot explain the whole immune resistance mechanisms that have different sides, including tumor cells, immune cells, and other cells in the tumor microenvironment. The primary aim of the current study is to study the effects of anti-PD-L1 blockade only on the tumor cell side. The increase in VEGFA expression of tumor cells treated with anti-PD-L1 antibodies may only partially explain the tumor cell side of the resistance mechanism. Further studies are needed to enlighten the role of anti-PD-L1 treatments on the VEGF pathway.

Loss of expression of E-cadherin is necessary to enable the tumor to progress and metastasize to distant organs in many tumors (Shields et al., 2019). Decreased expression of E-cadherin, a hallmark of EMT, is thought to play a key role in chemo-resistance development (Ribatti et al., 2020). However, the role of E-cadherin expression in resistance to immune checkpoint treatments is not known. The negative relation between PD-L1 and E-cadherin expression might explain the poor prognosis of cancer patients with high PD-L1 expression in previous reports (M. Zhang et al., 2017; W. Yu et al., 2020). Previous studies have shown that MDA-MB-231 cells lack E-cadherin expression (van de Wetering et al., 2001; Kenny et al., 2007; Eslami Amirabadi et al., 2019). Accordingly, we found no E-cadherin expression in MDA-MB-231 cells, following anti-PD-L1 antibody treatment, the E-cadherin expression levels were increased in PD-L1 high cells. Similar to the results of our study, it has been shown in a study that PD-L1 plays an active role in the expression of EMT-related molecules and that E-cadherin expression increases as a result of the knockdown of PD-L1 in lung cancer cells (Wendan Yu et al., 2020). Therefore, our results suggest that anti-PD-L1 antibody treatment in patients with PD-L1 high tumors not only activates the immune system but may also prevent metastasis by increasing E-cadherin expression. Interestingly, we found a significant reduction in E-cadherin expression levels in PD-L1 low tumor cells after anti-PD-L1 antibody treatment. In a study, it was determined that the downregulation of E-cadherin in prostate cancer cells increased EMT-mediated chemoresistance (Wang et al., 2017). Therefore, the relationship between PD-L1 and E-cadherin needs to be confirmed by further studies.

TGFβ1 is an immunosuppressive cytokine, which restricts T-cell infiltration to the tumor tissue in the tumor microenvironment. Inhibition of TGFβ1 causes a robust antitumor immune response and tumor regression (Holmgaard et al., 2018; Knudson et al., 2018; Mariathasan et al., 2018; Ravi et al., 2018; Lind et al., 2020). However, in our study, the anti-PD-L1 antibody treatment showed an increase in TGFβ1 expression in high PD-L1 expression tumor cells.

It has been shown that prostate cancers develop insensitivity to TGFβ to gain a growth advantage (Zhang et al., 2005). Likewise, LNCaP cells are reported to be sensitive to TGFβ, do not produce TGFβ and do not respond to TGFβ (Wilding et al., 1989). Similarly, we found no TGFβ1 gene expression in LNCaP cells; however, an increase in protein expression was observed at the 72nd hour of treatment, indicating that LNCaP cells may be sensitive to TGFβ after anti-PD-L1 antibody treatment. Although those findings do not provide a direct link between PD-L1 and TGFβ1 interaction, the combination of anti-PD-L1 treatment and anti-TGFβ1 could be an option in treating PD-L1 high tumors. Likewise, in a study by Holmgaard et al., it was found that the combination of inhibition of the TGFβ1 pathway and anti-PD-L1 therapy showed a strong anti-tumor efficacy, causing inhibition of tumor growth and complete regression in colon carcinoma models (Holmgaard et al., 2018). In another study, bifunctional blockade of PD-1/PD-L1 and TGF-β was shown to increase antitumor immune responses (Lind et al., 2020). On the other hand, we found that anti-PD-L1 antibody treatment reduces TGFβ1 expression in tumor cells with PD-L1 low expression. Although the findings obtained in this study do not provide a direct link between the PD-L1 and TGFβ1 interaction, further studies are needed as they show that there may be a relationship between them.

Overexpression of EGFR, a growth factor receptor tyrosine kinase and/or its ligand, is common in many types of cancer and supports the growth of solid tumors. Preclinical studies have shown that the EGFR signal acts on immune modulators by regulating MHC I/II and PD-L1 expressions on tumor cells and activation of lymphocytes (X. Li et al., 2018). In the current study, we found that anti-PD-L1 antibody treatment caused varying effects on PD-L1 high tumors. In our study, while the anti-PD-L1 antibody increased EGFR expression in MDA-MB-231 cells, it decreased EGFR expression in LNCaP cells. However, to further enlighten the role of PD-L1 expression on the EGFR pathway in cancer, the EGFR mutations that are most relevant in terms of tumor growth should also be studied. Furthermore, in our study, a decrease in EGFR expression was observed in PD-L1 low cells with anti-PD-L1 antibody treatment. Therefore, anti-PD-L1 antibody treatment in tumors with low PD-L1 seems to be advantageous in terms of increasing the survival of patients as it decreases EGFR expression. However, further research is needed.

Along with its proangiogenic effects, the increased bFGF may also induce an immunosuppressive milieu in the tumor microenvironment. Previous studies have shown that bFGF contributes to increased invasiveness of tumor cells by enhancing the ability of fibroblasts to produce various growth factors such as TGFβ1 and matrix metalloproteinase (MMP-9) (Korc and Friesel, 2009). The relation between PD-L1 and bFGF expression is not known. Studies have shown that LNCaP cells do not produce measurable amounts of bFGF (Nakamoto et al., 1992; Russell et al., 1999). In our study, in accordance with previous reports, bFGF expression was not detected in untreated LNCaP cells, while an increase in bFGF protein expression was detected after anti-PD-L1 treatment. This finding suggested that anti-PD-L1 treatment increased the expression of bFGF in LNCaP cells that do not express bFGF, suggesting that this increase in bFGF expression may be one of the mechanisms of immunotherapy resistance in prostate cancer cells with high PD-L1 expression.

## 5. Conclusion

Our results suggest that the binding of PD-L1 on tumor cells by an anti-PD-L1 monoclonal antibody may affect tumor intrinsic mechanisms. The activation of angiogenesis and metastasis-related pathways by anti-PD-L1 treatment in PD-L1 high tumors might be a tumor-promoting mechanism. The decrease of VEGFA, TGFβ1 and EGFR upon anti-PD-L1 treatment in PD-L1 low tumor cells provides a rationale for the use of those antibodies in PD-L1 low tumors. However, the findings obtained in this study need further confirmation by coculture with T cells and in vivo experiments.

## Supplementary

Supplementary Figure 1 PD-L1 expression of tumor cell lines. The representative histograms of PD-L1 expression in four tumor cells and the normalized median fluorescence intensity (MFI) (MFI of cells stained with specific MoAb/MFI of cells stained with isotype control).

Supplementary Figure 2 MTT assay after anti-PD-L1 antibody treatment. The anti-PD-L1 monoclonal antibody is not cytotoxic at applied doses (0.625–160 μg/mL), and cell viability is around 90%.

**Supplementary Table 1:** The sequences of primers and TaqMan probes were used in the qPCR.

Gene	Sequence
**VEGFA**	**F:** 5′-TGTGAATGCAGACCAAAGAAAGA-3′ **R:** 5′-GCTTTCTCCGCTCTGAGCAA-3′ **P:** 5′-AGAGCAAGACAAGAAAATCCCTGTGGGC-3′
**E-Cadherin**	**F:** 5′-TCCTGGGCAGAGTGAATTTT-3′ **R:** 5′-AGGCGTAGACCAAGAAATGG-3′ **P:** 5′-CCCTGCACACCCGATTCAAA-3′
**TGFβ1**	**F:** 5′-GTGGACATCAACGGGTTCACT-3′ **R:** 5′-TCCGTGGAGCTGAAGCAATAG-3′ **P:** 5′-CCGGCCTTTCCTGCTTCTCATGG-3′
**EGFR**	**F:** 5′-TCCCTCAGCCACCCATATGTAC-3′ **R:** 5′-GTCTCGGGCCATTTTGGAGAATTC-3′ **P:** 5′-ATCAACTCACGGAACTTTGGGCGACTATCTGCGTC-3′
**bFGF**	**F:** 5′-ACCCCGACGGCCGA-3′ **R:** 5′-TCTTCTGCTTGAAGTTGTAGCTTGA-3′ **P:** 5′-TCCGGGAGAAGAGCGACCCTCAC-3′
**b-actin**	**F:** 5′-CCCTGGAGAAGAGCTACGAG-3′ **R:** 5′-AGGCGTAGACCAAGAAATGG-3′ **P:** 5′-CCCTGCACACCCGATTCAAA-3′

**Supplementary Table 2:** Gene expressions at 72 hours of IgG1 isotypic antibody treatment of PD-L1 high and PD-L1 low cells (*p ≤ 0.05, **p ≤ 0.001; compared to control).

VEGFA	IgG1 Isotype Control (Relative ratio ± SD)			
	40	20	2	Control
MDA-MB-231	0.68 ± 0.01**	0.30 ± 0.00**	1.02 ± 0.01	1.00 ± 0.07
LNCaP	0.90 ± 0.05	1.07 ± 0.06	1.01 ± 0.08	1.00 ± 0.05
DU145	2.03 ± 0.05**	1.41 ± 0.06**	1.27 ± 0.04*	1.00 ± 0.04
E-Cadherin	IgG1 Isotype Control (Relative ratio ± SD)			
	40	20	2	Control
LNCaP	1.06 ± 0.05	0.98 ± 0.07	1.05 ± 0.06	1.03 ± 0.04
TGFb1	IgG1 Isotype Control (Relative ratio ± SD)			
	40	20	2	Control
MDA-MB-231	0.73 ± 0.06*	1.11 ± 0.06	1.10 ± 0.09	1.01 ± 0.12
DU145	1.09 ± 0.23	1.20 ± 0.23	1.09 ± 0.17	1.18 ± 0.43
EGFR	IgG1 Isotype Control (Relative ratio ± SD)			
	40	20	2	Control
MDA-MB-231	0.89 ± 0.03	1.10 ± 0.07	1.34 ± 0.01**	1.01 ± 0.07
LNCaP	0.73 ± 0.10	1.25 ± 0.17	1.34 ± 0.02	1.01 ± 0.07
MDA-MB-435	0.87 ± 0.08	0.96 ± 0.05	0.97 ± 0.38	1.01 ± 0.08

## Figures and Tables

**Table 1:** Quantification of proteins at 72 h after anti-PD-L1 MoAb and IgG1 isotypic control treatment in tumor cells with high PD-L1 expression levels (*p ≤ 0.05; compared to 0 mg/mL dose level).

LNCaP				
VEGFA/β-actin ± SD				
	40	20	2	0
Anti-PD-L1	1.2 ± 0.01	2.77 ± 0.044*	2.12 ± 0.01*	1 ± 0.017
IgG1	1.15 ± 0.012	1.35 ± 0.046	1.11 ± 0.012	1 ± 0.01
E-Cad/β-actin ± SD				
	40	20	2	0
Anti-PD-L1	1.68 ± 0.026*	1.80 ± 0.017*	2.23 ± 0.02*	1 ± 0.01
IgG1	1.08 ± 0.015	1.29 ± 0.012	1 ± 0.05	1 ± 0.01
TGFβ1/β-actin ± SD				
	40	20	2	0
Anti-PD-L1	0.8 ± 0.02	1.14 ± 0.032*	2.26 ± 0.015*	1 ± 0.017
IgG1	1.08 ± 0.021	1.07 ± 0.01	1.08 ± 0.02	1 ± 0.052
EGFR/β-actin ± SD				
	40	20	2	0
Anti-PD-L1	0.58 ± 0.015	0.49 ± 0.01	0.43 ± 0.021	1± 0.01
IgG1	1.79 ± 0.015	1.32 ± 0.026	1.04 ± 0.01	1 ± 0.02
bFGF/β-actin ± SD				
	40	20	2	0
Anti-PD-L1	1.48 ± 0.012	2.15 ± 0.01*	1.64 ± 0.01	1 ± 0.017
IgG1	1.03 ± 0.021	0.98 ± 0.01	1.1 ± 0.01	1 ± 0.026

MDA-MB-231				
VEGFA/β-actin ± SD				
	40	20	2	0
Anti-PD-L1	4.05 ± 0.06*	2.11 ± 0.035*	1.54 ± 0.071	1 ± 0.02
IgG1	1 ± 0.049	0.97 ± 0.042	1.07 ± 0.025	1 ± 0.026
E-Cad/β-actin ± SD				
	40	20	2	0
Anti-PD-L1	1.40 ± 0.208*	1.14 ± 0.123	1.08 ± 0.021	1 ± 0.085
IgG1	0.89 ± 0.015	1.01 ± 0.01	0.77 ± 0.021	1 ± 0.02
TGFβ1/β-actin ± SD				
	40	20	2	0
Anti-PD-L1	1.87 ± 0.02*	1.63 ± 0.021	1.22 ± 0.026	1 ± 0.044
IgG1	1.06 ± 0.053	0.89 ± 0.025	1.08 ± 0.015	1 ± 0.017
EGFR/β-actin ± SD				
	40	20	2	0
Anti-PD-L1	1.23 ± 0.03	2.03 ± 0.064	1.65 ± 0.015	1 ± 0.017
IgG1	0.69 ± 0.036	1.47 ± 0.03	1.47 ± 0.026	1 ± 0.03
bFGF/β-actin ± SD				
	40	20	2	0
Anti-PD-L1	1.11 ± 0.085	1.11 ± 0.159	1.12 ± 0.01	1 ± 0.05
IgG1	1.06 ± 0.036	0.92 ± 0.031	1.08 ± 0.021	1 ± 0.044

**Table 2:** Secreted protein levels at 72 h after anti-PD-L1 antibody treatment in tumor cells with high PD-L1 expression levels (*p ≤ 0.05 **p ≤ 0.01; compared to 0 mg/mL dose level).

	VEGFA (pg/mL ± SD)			
	40	20	2	0 (μg/mL)
MDA-MB-231	5293.2 ± 14.5	5287.2 ± 66.3	5182.1 ± 45.7	5118.2 ± 70.2
LNCaP	1424.5 ± 84.22	1762.6 ± 10.85	1803.5 ± 1.37	1705.9 ± 64.9
	E-Cadherin (ng/mL ± SD)			
	40	20	2	0 (μg/mL)
MDA-MB-231	0.85 ± 0.08*	0.70 ± 0.02	0.63 ± 0.02	0.67 ± 0.02
LNCaP	0.49 ± 0.02**	0.37 ± 0.01	0.42 ± 0.001	0.26 ± 0.003
	TGFβ1 (pg/mL ± SD)			
	40	20	2	0 (μg/mL)
MDA-MB-231	1380.5 ± 75.7	863.6 ± 14.8	1137.4 ± 34.8	1285.4 ± 86.01
LNCaP	448.8 ± 3.1	469.6 ± 20.1	478.1 ± 44.1	455.5 ± 21.2
	EGFR (ng/mL ± SD)			
	40	20	2	0 (μg/mL)
MDA-MB-231	34.22 ± 1.22*	32.5 ± 0.16*	26.14 ± 0.35	26.9 ± 2.41
LNCaP	0.93 ± 0.017	0.88 ± 0.045	0.73 ± 0.102*	1.072 ± 0.011
	bFGF (pg/mL ± SD)			
	40	20	2	0 (μg/mL)
LNCaP	15.54 ± 0.6	21.64 ± 1.38*	14.05 ± 1.64	12.78 ± 0.05

**Table 3:** Quantification of proteins at 72 h after anti-PD-L1 MoAb and IgG1 isotypic control treatment in tumor cells with low PD-L1 expression levels (*p ≤ 0.05; compared to 0 mg/mL dose level).

DU145				
TGFβ1/β-actin ± SD				
	40	20	2	0
Anti-PD-L1	0.8 ± 0.02*	77 ± 0.021*	0.88 ± 0.02	1 ± 0.1
IgG1	1.15 ± 0.015	0.97 ± 0.01	0.98 ± 0.021	1 ± 0.044
				
VEGFA/β-actin ± SD				
	40	20	2	0
Anti-PD-L1	1.6 ± 0.2*	1.37 ± 0.006*	1.01 ± 0.01	1 ± 0.01
IgG1	0.04 ± 0.02	1.81 ± 0.021	0.71 ± 0.015	1 ± 0.017
				
E-Card/β-actin ± SD				
	40	20	2	0
Anti-PD-L1	1.54 ± 0.017*	1.63 ± 0.021	1.70 ± 0.02	1 ± 0.01
IgG1	1.1 ± 0.01	0.17± 0.025	1.91 ± 0.021	1 ± 0.044
MDA-MB-435				
EGFR/β-actin ± SD				
	40	20	2	0
Anti-PD-L1	0.8 ± 0.01	1.05 ± 0.015	1.65 ± 0.015	1 ± 0.026
IgG1	1.37 ± 0.026	1.1 ± 0.026	0.77 ± 0.01	1 ± 0.055

**Table 4:** Secreted protein levels at 72 h after anti-PD-L1 antibody treatment in tumor cells with low PD-L1 expression levels (*p ≤ 0.05, **p ≤ 0.01; compared to 0 mg/mL dose level).

DU145			
VEGFA (pg/mL ± SD)			
40	20	2	0 (μg/mL)
4590.9 ± 1.03	3394.3 ± 143.8*	4366.5 ± 45.01	4346.7 ± 163.7
E-Cadherin (ng/mL ± SD)			
40	20	2	0 (μg/mL)
2.76 ± 0.19	0.91 ± 0.11*	2.97 ± 0.24	2.55 ± 0.58
TGFβ1 (pg/mL ± SD)			
40	20	2	0 (μg/mL)
1661.3 ± 38.2	702.7 ± 80.4**	1477.1 ± 26.4	1575.5 ± 35.2
MDA-MB-435			
EGFR (ng/mL ± SD)			
40	20	2	0 (μg/mL)
19.98 ± 1.17	21.36 ± 0.64	18.48 ± 0.41	22.86 ± 3.46
