# Supplementary figures and images for: The effects of anti-PD-L1 monoclonal antibody on the expression of angiogenesis and invasion-related genes
Source: Turk J Biol. 2023 Jun 7;47(4):262–75. doi: 10.55730/1300-0152.2661 (PMC10751090; doi:10.55730/1300-0152.2661)

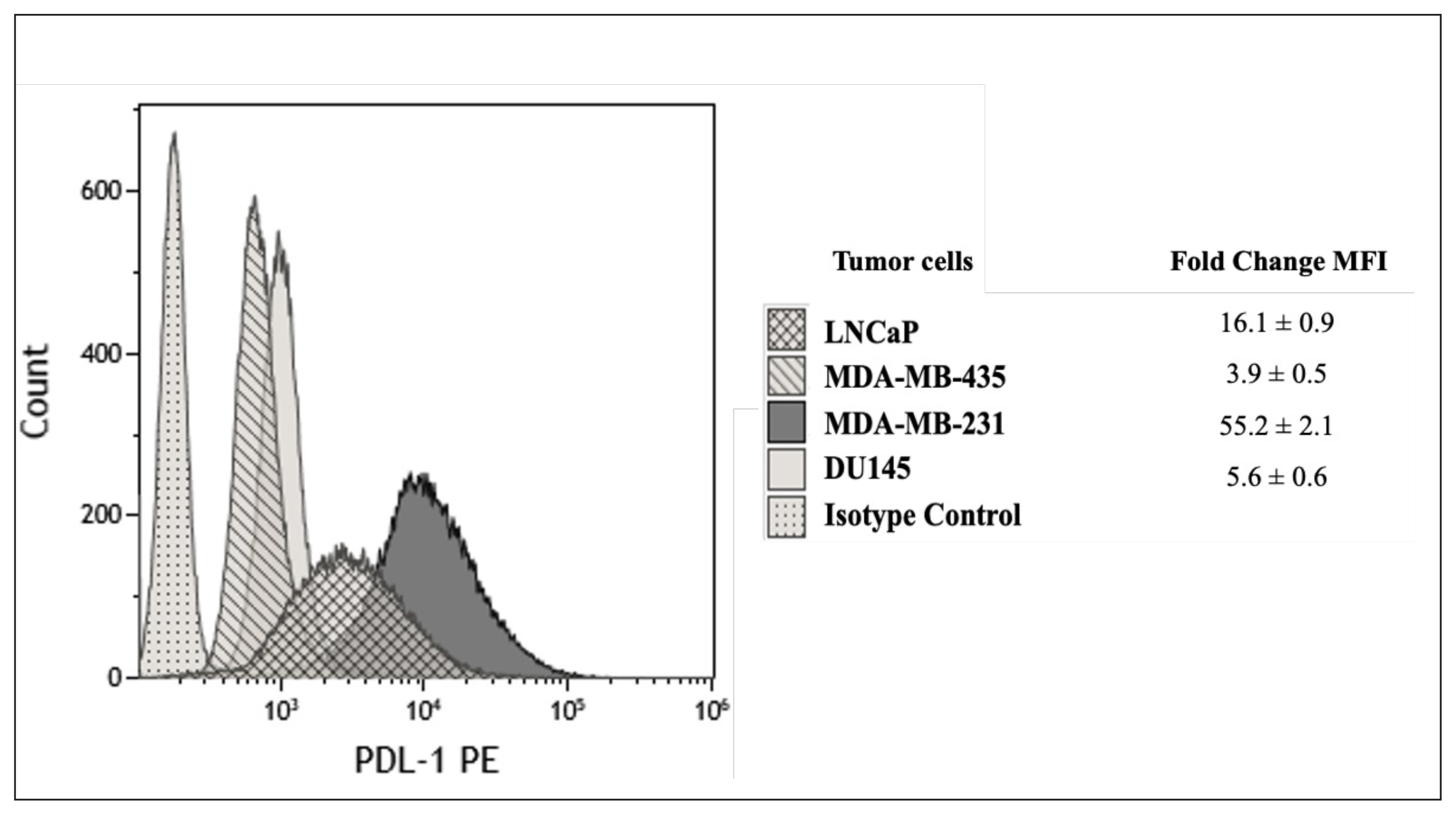

Supplement: Supplementary Figure 1 — PD-L1 expression of tumor cell lines. The representative histograms of PD-L1 expression in four tumor cells and the normalized median fluorescence intensity (MFI) (MFI of cells stained with specific MoAb/MFI of cells stained with isotype control). [file turkjbiol-47-4-262s1.tif]

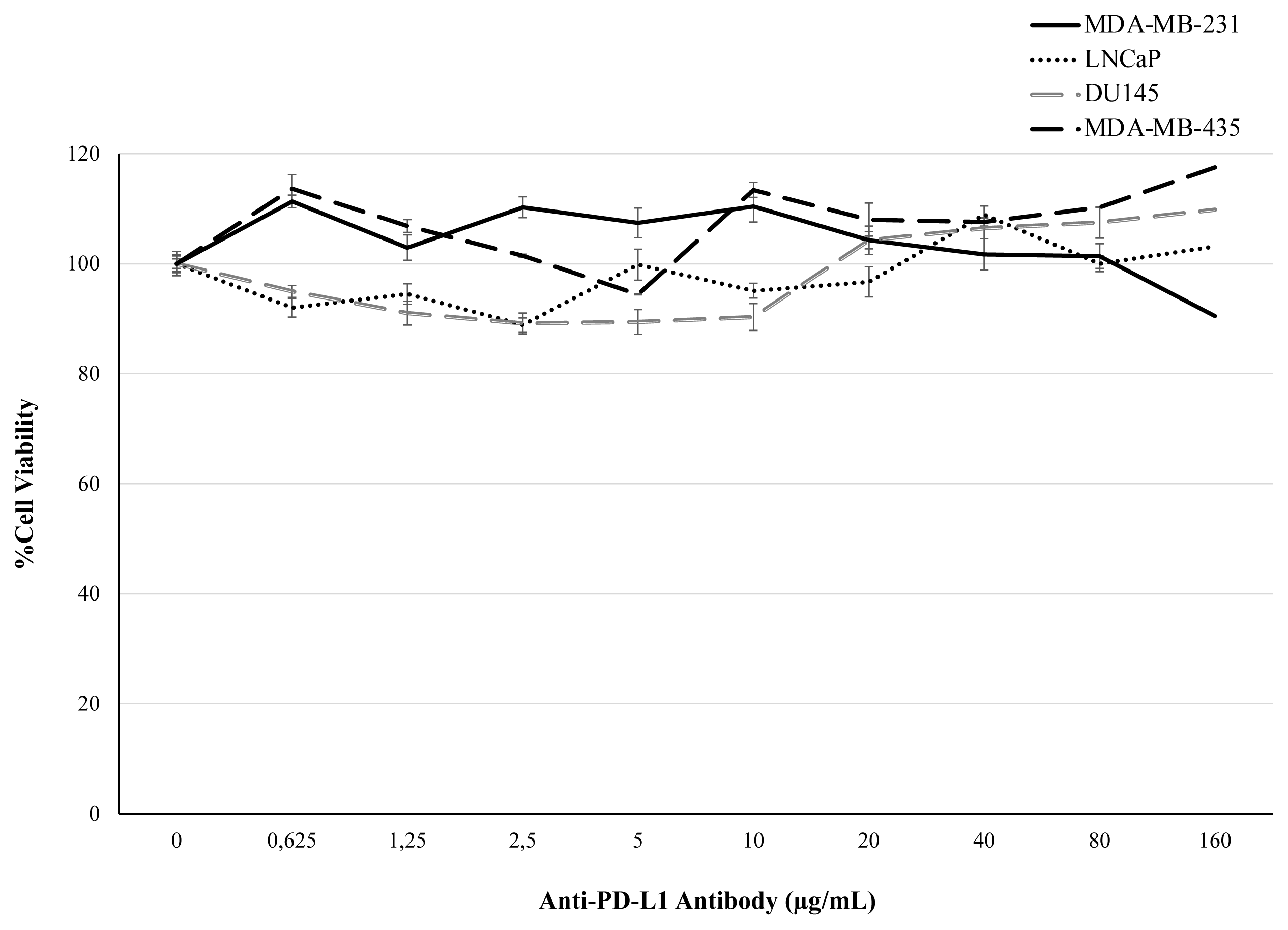

Supplement: Supplementary Figure 2 — MTT assay after anti-PD-L1 antibody treatment. The anti-PD-L1 monoclonal antibody is not cytotoxic at applied doses (0.625–160 μg/mL), and cell viability is around 90%. [file turkjbiol-47-4-262s2.tif]
